# SITC perspective: leveraging patient enrichment biomarkers to accelerate early phase IO drug development

**DOI:** 10.1136/jitc-2024-010739

**Published:** 2025-06-22

**Authors:** Leisha A Emens, Christine Moussion, Patrick Hwu, James L Gulley, Pamela S Ohashi, Carlo B Bifulco, David Feltquate

**Affiliations:** 1 Kaiser Permanente, South Sacramento, California, USA; 2 Genentech, South San Francisco, California, USA; 3 H. Lee Moffitt Cancer Center and Research Institute, Tampa, Florida, USA; 4 NCI, National Institutes of Health, Bethesda, Maryland, USA; 5 Princess Margaret Hospital Cancer Centre, Toronto, Ontario, Canada; 6 Department of Immunology, University of Toronto, Toronto, Ontario, Canada; 7 Providence Portland Medical Center, Earle A. Chiles Research institute, Portland, Oregon, USA; 8 iTeos Therapeutics Inc, Watertown, Massachusetts, USA

**Keywords:** Biomarker, Biopsy, Tumor Microenvironment, Next generation sequencing - NGS, Pathology

## Abstract

Cancer immunotherapy (IO) enables patients to live well with cancer for many years, or even be cured. Several investigational IO agents recently failed in early-phase or late-phase trials, leading some to doubt the future of IO. Patient heterogeneity (eg, tumor characteristics, treatment history) increases the risk that a clinically active IO drug might be discarded. Enriching enrollment for patients with biomarkers hypothesized to reflect a higher probability of clinical benefit across clinical development should mitigate this risk. The Society for Immunotherapy of Cancer convened diverse IO stakeholders to discuss leveraging biomarkers at the earliest stages of drug development to accelerate the delivery of innovative IO agents to patients. This group developed a framework based on a biomarker-based enrichment strategy in early trials that evolves into the development of more precise predictive biomarkers in late phase trials. This framework integrates mechanistic insights related to the drug and its impact on the tumor microenvironment derived from preclinical data, digital pathology, exploratory multiomics, and artificial intelligence that are continuously refined through both adaptive and randomized clinical trials. Biomarker-based enrichment in early clinical development should de-risk late-stage trials, ultimately expanding the portfolio of innovative IO drugs available to patients.

 The first wave of immunotherapy (IO)—especially immune checkpoint inhibitors (ICIs) and chimeric antigen receptor T-cell therapy (CAR-T)—transformed cancer care by delivering long-term clinical benefit and even cure to some patients.^1^ However, not all patients with cancer are eligible for these treatments, and primary or acquired drug resistance to current agents may occur.^2^ Novel IO drugs that overcome these limitations and expand the benefit of IO to more patients are urgently needed. In 2024, the Society for Immunotherapy of Cancer (SITC) established a goal of 100 new, unique IO approvals in the next decade. Supporting this goal, SITC strongly encourages biomarker-based enrichment strategies to support the enrollment of patients more likely to respond in early phase clinical trials of emerging novel IO agents.

Over the last several years, efforts to generate a second wave of innovative IO drugs specific for novel immune targets and pathways have not often detected meaningful clinical signals, leading to skepticism from some stakeholders. In July 2024, SITC convened a diverse group of experts in IO—representing patients with cancer, translational researchers, clinicians, pathologists, experts in artificial intelligence, and professionals in academia and biotech/pharma—to discuss potential strategies for effectively leveraging biomarkers to accelerate IO drug development and catalyze the next wave of clinically transformative IO strategies (list of participants in online supplemental table 1). We also engaged regulatory colleagues for their perspective. The convened group agreed that patients enrolled in early phase IO clinical trials are typically heterogeneous due to both varying tumor biology and prior treatment history—they may be IO naive or experienced, and they may also have primary or acquired drug resistance. Due to this heterogeneity, only a very small number of enrolled patients may have the potential to respond to the investigational drug. This dilution of patients with potential to respond creates a significant risk of a false negative clinical signal, leading to the inadvertent discarding of a drug with clinical activity. For example, testing an anaplastic lymphoma kinase (ALK) inhibitor in a small cohort of unselected patients with non-small cell lung cancer, where the prevalence of an ALK alteration is 3–5%, would almost certainly fail to detect a clinical signal.^3^ To both mitigate the risk of missing a clinical signal and accelerate the pace of IO drug development, we propose developing a biomarker strategy for patient enrichment in the earliest clinical trials of new agents, thus increasing the likelihood of detecting a clinical signal. The level of enrichment would be determined by the sponsor depending on the preclinical data, trial design and cohort size. For example, a sponsor may design the study such that 65% of patients express the biomarker, and 35% do not. Alternatively, a sponsor may require biomarker expression initially in all enrolled patients, and if a clinical signal is detected, adapt the design to include patients with lower or no expression of the biomarker to explore other patient populations. A successful patient enrichment strategy will by design be less specific (table 1) than a strategy using defined predictive biomarkers for more stringent patient selection. However, in addition to increasing the likelihood of detecting a clinical signal, it should lay the critical scientific groundwork for retrospectively identifying more specific predictive biomarkers of response, resistance, and toxicity for use in patient selection for prospective pivotal clinical trials and future clinical decision-making.

**Table 1:** Proposed profile of an enrichment biomarker for signal detection in early phase trials

Assay performance	Assay sensitivity/specificity sufficient to identify relevant biology
Enrichment features	Level of hypothesized enrichment of twofold to threefold or more Extent of enrichment likely to be context-specific
Operating characteristics	Low inter-laboratory and inter-reader variability
Resources considerations	Low assay cost and turn-around time

Most current immunotherapies—ICIs, CAR-T, tumor infiltrating lymphocytes (TIL), engineered T-cell receptor T cells (TCR-T), and T-cell engagers—are based on directly manipulating T-cell biology. Consistent with this, established predictive biomarkers for patient selection—programmed cell death ligand 1 (PD-L1), microsatellite instability (MSI)-high, DNA mismatch repair deficiency (dMMR), and tumor mutational burden (TMB)—reflect a connection to T-cell biology.^4^ For IO agents highly dependent on T cells, a patient enrichment strategy might be a simple immunohistochemistry (IHC) stain for CD8. Such an IHC stain would also provide basic insight into the spatial architecture of the tumor microenvironment (TME), such as whether it has an inflamed, excluded, or desert immuno-phenotype. If a CD8 enrichment strategy supported the detection of a clinical signal, retrospective studies using multiomics could identify a biomarker profile more precisely defining T-cell subpopulations and/or spatial organization associated with the clinical activity of the new investigational agent. A recent study conducted an unbiased analysis of genomics, transcriptomics, and clinical data of a large patient cohort (n=479) with a range of metastatic cancers treated with ICIs.^5^ The data revealed five independent biomarkers of response and survival relevant to all tumor types: any prior treatment, tumor proliferative potential, T-cell infiltration, TMB, and transforming growth factor-β activity in the TME; these findings were validated in six independent patient cohorts (n=1,491). The authors suggest that these five biomarkers provide a frame of reference for organizing established and emergent knowledge about predictive biomarkers of clinical benefit from ICI treatment.

It is equally important to identify predictive biomarkers of toxicity and long-term clinical benefit to accelerate the delivery of transformative IO medicines to patients. There are currently no approved predictive biomarkers of IO drug toxicity. Identifying these biomarkers as drug-related adverse events emerge will facilitate the inclusion of patients most likely to benefit from a new IO drug with the least incurred toxicity cost. In addition, clinical endpoints like overall response rate(ORR) or progression-free survival (PFS) are most often based on the measurement of tumor burden by imaging, but the gold standard endpoint for regulatory approval in the USA is overall survival (OS). In a step forward, minimal residual disease (MRD) was accepted in 2024 as a surrogate endpoint of PFS and OS for accelerated regulatory approval in multiple myeloma.^6^ In contrast, despite initial interest in the value of pathologic complete response (pCR) in predicting long-term clinical benefit in breast cancer, this association only holds up at the patient level, not at the trial level.^7^ This remains a topic of debate. SITC strongly encourages the evaluation of surrogate measures of long-term clinical benefit using radiomics, pathologic response, and/or blood-based biomarkers such as circulating tumor DNA (ctDNA) or circulating tumor cells (CTCs) (or others) as early in clinical development as possible.

In contrast to agents more dependent on T-cell activity, many emerging innovative IO agents target alternative/new regulatory pathways or cell types/structures beyond T cells, while still influencing T-cell activity. Examples of these alternative targets include tumor-associated tertiary lymphoid structures (TLS—detected by H&E and CD20+B cells)^8 9^; myeloid cells: tumor-associated macrophages (TAMs)^10^ and myeloid-derived suppressor cells (MDSCs), which are quite heterogenous due to functional and phenotypic plasticity (most commonly assessed by CD68 and CD163)^11 12^; neutrophils: heterogenous cells that share some common regulatory pathways with other myeloid cells, with an elevated peripheral blood neutrophil to lymphocyte ratio (NLR) associated with poor survival^13^; and fibroblasts/fibrosis, which have historically been difficult to measure and characterize at the molecular level.^14 15^ Cytokine pathways (such as IL-6,^16^ CXCL9/CXCL10-CXCR3^17^) and natural killer (NK) cells^18^ should also be considered as appropriate. A biomarker that enriches for non-T-cell biology in early phase trials should be based on preclinical data that demonstrates pathway modulation relative to baseline, ideally in the context of tumor regression in preclinical or clinical models.

From an operational perspective, the specimen quantity, quality, type, and processing time/conditions impact the results of tissue analysis.^19^ In general for the TME, at least three to five core biopsies or an excisional biopsy are best for assessment of the tumor architecture and infiltrating immune cells. Fine needle aspiration biopsies are to be discouraged, as the cells obtained are dispersed, and do not accurately reflect the TME architecture. The optimal number and location/distribution of core biopsies is not well-studied, and consensus is needed. Digital pathology is emerging as a powerful technology for quantitating immune cells, though standardized algorithms for digitizing pathology images are needed. Finally, artificial intelligence/deep learning models are emerging that can simplify and standardize the reading of pathologic images. These will need broader implementation.

Given this current landscape, what is the best way to proceed with a biomarker enrichment strategy for early phase trials of novel IO agents? The potential advantages are clear. First, the enrichment strategy would increase the likelihood of detecting a clinical signal. Second, a successful patient enrichment strategy should set the stage for retrospective multiomics studies designed to identify a more precise predictive biomarker for patient selection to be used in pivotal studies and future clinical care. Third, it would decrease the enrollment of patients less likely to benefit. The challenges are also clear. First, given the diversity and dynamic nature of the antitumor immune response, the enrichment strategy will have to be customized to the context of the drug and pathway under study—it will not be one size fits all. Second, many of the most clinically relevant markers of cell types and regulatory pathways that modulate antitumor immunity remain to be elucidated. Given these current constraints, we propose implementing a patient enrichment strategy in early phase IO trials based on biomarkers characterized at baseline and in the context of pharmacodynamic changes in the TME that occur with drug exposure and are associated with tumor regression in preclinical and/or clinical models (Phase 0 trials). If this leads to the detection of a clinical signal in patients, adaptive clinical protocols may further refine the biomarker and its cut-offs based on incoming data. Moreover, associated baseline and on-treatment tumor biopsies from patients exposed to the drug can be deeply interrogated with multiomics technologies and advanced data analysis protocols both to further define immunobiology, and to identify refined biomarkers for both enrichment and more specific patient selection.

SITC leadership has prioritized several initiatives to support the field in developing biomarkers for both patient enrichment and more precise patient selection. We will hold a workshop in 2025 focused on the diverse cell types important in the antitumor immune response, and sophisticated technologies such as digital pathology, molecular imaging, radiomics, and artificial intelligence/deep learning that can support and accelerate biomarker development. We will publish consensus manuscripts focused on the definition of TLS, and establishing standard algorithms for digital pathology. We will explore public/private partnerships and other avenues to facilitate the retrospective analysis of archival clinical trial biospecimens for novel IO agents that target promising pathways but failed to demonstrate clinical activity. Collaborative models involving academia, industry, and regulatory bodies, such as pre-competitive consortia to share biomarker data and establish standardized assays, will almost certainly accelerate biomarker development. We will also maintain an active dialogue with regulatory colleagues about the best use of biomarkers for patient enrichment and selection that reflect the clinical activity and/or toxicity associated with novel IO drugs. It is notable that most currently approved predictive biomarkers are based on genomic analyses, with only one based on protein expression by IHC (PD-L1). As novel biomarkers and technologies for measuring them emerge, SITC is committed to assembling key stakeholders to develop field-wide consensus and facilitate the development of guidelines about what novel assays (flow cytometry, cytokine-based, ctDNA, CTCs) should demonstrate to obtain regulatory approval. In parallel, SITC strongly supports the continued development of early surrogate biomarkers for OS (MRD, ctDNA, pCR), and will work with regulators and other stakeholders to guide their implementation. By working together, we will continue to realize the promise of immunotherapy by generating the best new IO drugs in order to deliver the right drug to the right patient at the right time.

## Supplementary material

10.1136/jitc-2024-010739 online supplemental file 1
