# Supplementary material for: SITC perspective: leveraging patient enrichment biomarkers to accelerate early phase IO drug development
Source: J Immunother Cancer. 2025 Jun 22;13(6):e010739. doi: 10.1136/jitc-2024-010739 (PMC12184391; doi:10.1136/jitc-2024-010739)
Supplement: online supplemental file 1 [file jitc-13-6-s001.docx]

**Table S1** Society for Immunotherapy of Cancer 2024 Strategic Retreat Attendees

| **Name** | **Affiliation** |
| --- | --- |
| Arlene H. Sharpe, MD PhD FAIO | Harvard Medical School |
| Avery D. Posey, Jr., PhD | University of Pennsylvania Perelman School of Medicine |
| Bernard A. Fox, PhD | Earle A. Chiles Research Institute, |
| Carlo Bifulco, MD | Providence Genomics |
| Charles Drake, MD, PhD | Janssen R&D |
| Christine Moussion, PhD | Genentech |
| Claire I. Vanpouille-Box, PhD | Weill Cornell Medicine |
| Daniel Powell, PhD | University of Pennsylvania |
| Daniel S. Chen, MD, PhD | Synthetic Design Lab Inc. |
| David Feltquate, MD PhD | iTEOS Therapeutics |
| Elliot Chartash, MD | Merck Sharp & Dhome LLC |
| Harriet Kluger, MD | Yale University |
| James L. Gulley, MD, PhD, FACP | National Cancer Institute |
| Jane Perlmutter, PhD, MBA | Gemini Group |
| Jason J. Luke, MD, FACP | UPMC Hillman Cancer Center |
| Jennifer L. Guerriero, PhD | Brigham and Women's Hospital |
| Kimberly Schluns, MD, PhD | Kite Pharma |
| Lawrence Fong, MD | Fred Hutchinson Cancer Center |
| Leisha A. Emens, MD, PhD | Kaiser Permanente |
| Marc S. Ernstoff, MD | NIH/NCI/DCTD/DTP-IOB |
| Mario Sznol, MD | Yale School of Medicine |
| Michael T. Lotze, MD, FACS | University of Pittsburgh |
| Pamela S. Ohashi, PhD | Princess Margaret Cancer Centre |
| Patrick Hwu, MD | Moffitt Cancer Center |
| Raj Puri, MD, PhD | Iovance Biotherapeutics, Inc. |
| Rajaa Nahra, MD, MSc | GSK |
| Ryan J. Sullivan, MD | Harvard Medical School, Massachusetts General Hospital |
| Saman Maleki, PhD | Western University |
| Sarah Warren, PhD | Kite, A Gilead Company |
| Scott Rodig, MD, PhD | Brigham & Women's Hospital, Inc. |
| Siraj Ali, MD, PhD | Lunit Inc. |
| Stephen Lim, MD | Novartis |
| Tabetha Sundin PhD, HCLD, MB | AstraZeneca |
| Thomas F. Gajewski, MD, PhD | University of Chicago |
| Tullia C. Bruno, PhD | University of Pittsburgh |
